# Supplementary material for: Is There an Association between Paw Preference and Emotionality in Pet Dogs?
Source: Animals (Basel). 2022 Apr 29;12(9):1153. doi: 10.3390/ani12091153 (PMC9103732; doi:10.3390/ani12091153)
Supplement: Supplementary file 1 [file animals-12-01153-s001.zip › Text S1_Written instructions for the paw preference tests (for dog owners).pdf]

Text S1: Written instructions for the paw preference tests (for dog owners)

Kong™ Test

This test assesses which paw your dog preferentially uses to hold a food-stuffed Kong™.

Preparation

1. Fill a Kong™ with wet dog food (if you have 2 Kong™ toys of the same size, prepare 2 Kong™ toys).
2. Look for a quiet place where your dog can eat from the Kong™ without being distracted. It is important that your dog can move as freely as possible. Make sure that your dog has enough appetite - do not do this test just after they have had dinner. You need paper and pencil.

Test

1. Ask your dog to lie down and put a filled Kong™ between both front legs of your dog.
2. Now, the dog is allowed to eat from the Kong™. Use a tally list to note down which paw the dog uses each time they hold the Kong™. Only the right and left front paw are counted. The simultaneous use of both paws to hold the Kong™ is not counted.
3. **If your dog has ever shown any signs of aggression towards you or another family member, especially in contexts of food or toys, be careful and do not remove the Kong™ when the dog is still eating from it**.
4. In total, 50 paw uses are needed. If all the food has been eaten from the Kong™, provide the second Kong/toy or refill the empty Kong™.
5. Note the size of your Kong™.
6. Please see the video below for a guide.

Score for the Kong™ test. Please indicate how many times each paw was used to hold the Kong™ for up to a total of 50 incidents of toy holding.

Left paw: ___ Right paw: ___

Size of Kong™

- S (7cm)
- M (8.5 cm)
- L (10.5 cm)
- XL (12.5 cm)
- XXL (15 cm)

First Step Test

This test assesses which paw your dog uses first when moving after being stationary.

Preparation

1. Look for a quiet place where your dog is not distracted by external influences (other people, animals or noises).

Test

1. Your dog needs to **stand** (front and hind paws parallel to each other).
2. Squat down in a straight line in front of your dog (distance approx. 2 m).
3. Call your dog and note which front paw your dog uses first when they start moving toward you.
4. Repeat the procedure 4 more times (5x in total)
5. Repeat the procedure with other stationary positions, i.e. **sitting** (5x) and **lying** (5x).
6. Please see the video below for a guide.

Score for the First Step Test from a standing position. Please indicate how many times each paw was used first during the 5 trials.

Left paw: ___ Right paw: ___

Score for the First Step Test from a sitting position. Please indicate how many times each paw was used first during the 5 trials.

Left paw: ___ Right paw: ___

Score for the First Step Test from a lying position. Please indicate how many times each paw was used first during the 5 trials.

Left paw: ___ Right paw: ___

Hurdle Test

This test assesses which paw your dog uses first when stepping over a hurdle.

Preparation

1. Look for a quiet place where your dog is not distracted by external influences (e.g., other people, animals or noises).
2. Build a hurdle (e.g., out of a broom and books; see video). The height of the hurdle should be approx. half the length of the dog's front leg.

Test

1. Your dog needs to **stand** in front of the hurdle (both front and hind paws parallel to each other). The distance between your dog and the hurdle should be such that it can step over the hurdle without any intermediate steps.
2. Squat down directly facing your dog, about 2 m away, on the other side of the hurdle.
3. Call your dog and note which front paw it uses first when stepping over the hurdle.
4. Repeat the procedure 4 more times (5x in total).
5. Repeat the procedure when your dog is **sitting** (5x) and **lying** (5x) in front of the hurdle.
6. Please see the video below for a guide.

Score for the Hurdle Test from a <u>standing position</u>. Please indicate how many times each paw was used first during the 5 trials.

Left paw: ___ Right paw: ___

Score for the Hurdle Test from a <u>sitting position</u>. Please indicate how many times each paw was used first during the 5 trials.

Left paw: ___ Right paw: ___

Score for the Hurdle Test from a <u>lying position</u>. Please indicate how many times each paw was used first during the 5 trials.

Left paw: ___ Right paw: ___
